# Two-photon polymerized “nichoid” substrates maintain function of pluripotent stem cells when expanded under feeder-free conditions

**DOI:** 10.1186/s13287-016-0387-z

**Published:** 2016-09-09

**Authors:** Michele M. Nava, Alessio Piuma, Marina Figliuzzi, Irene Cattaneo, Barbara Bonandrini, Tommaso Zandrini, Giulio Cerullo, Roberto Osellame, Andrea Remuzzi, Manuela T. Raimondi

**Affiliations:** 1 Department of Chemistry, Materials and Chemical Engineering “Giulio Natta”, Politecnico di Milano, 32, piazza Leonardo da Vinci, 20133 Milan, Italy; 2 IRCCS Istituto di Ricerche Farmacologiche “Mario Negri”, Bergamo, Italy; 3 Istituto di Fotonica e Nanotecnologie (IFN) - CNR and Department of Physics, Politecnico di Milano, Milan, Italy; 4 Department of Management, Information and Production Engineering, University of Bergamo, Dalmine, Italy

**Keywords:** Nanofabrication, Two-photon polymerization, Embryonic stem cells, Pluripotency, Scaffold, Mechanobiology

## Abstract

**Background:**

The use of pluripotent cells in stem cell therapy has major limitations, mainly related to the high costs and risks of exogenous conditioning and the use of feeder layers during cell expansion passages.

**Methods:**

We developed an innovative three-dimensional culture substrate made of “nichoid” microstructures, nanoengineered via two-photon laser polymerization. The nichoids limit the dimension of the adhering embryoid bodies during expansion, by counteracting cell migration between adjacent units of the substrate by its microarchitecture. We expanded mouse embryonic stem cells on the nichoid for 2 weeks. We compared the expression of pluripotency and differentiation markers induced in cells with that induced by flat substrates and by a culture layer made of kidney-derived extracellular matrix.

**Results:**

The nichoid was found to be the only substrate, among those tested, that maintained the expression of the OCT4 pluripotency marker switched on and, simultaneously, the expression of the differentiation markers GATA4 and α-SMA switched off. The nichoid promotes pluripotency maintenance of embryonic stem cells during expansion, in the absence of a feeder layer and exogenous conditioning factors, such as the leukocyte inhibitory factor.

**Conclusions:**

We hypothesized that the nichoid microstructures induce a genetic reprogramming of cells by controlling their cytoskeletal tension. Further studies are necessary to understand the exact mechanism by which the physical constraint provided by the nichoid architecture is responsible for cell reprogramming. The nichoid may help elucidate mechanisms of pluripotency maintenance, while potentially cutting the costs and risks of both feed-conditioning and exogenous conditioning for industrial-scale expansion of stem cells.

**Electronic supplementary material:**

The online version of this article (doi:10.1186/s13287-016-0387-z) contains supplementary material, which is available to authorized users.

## Background

Cell-based therapies represent an important strategy to restore the function of injured cells, tissues, and organs. However, the limited availability of functional cells has hampered the success of this strategy. The recent advances in stem cell biology offer an opportunity to address this challenge. Embryonic stem (ES) cells have a great potential because they can proliferate indefinitely and, at the same time, retain the developing potential to generate cells of all three embryonic germ layers [32]. However, ES cells do not maintain their pluripotency during expansion passages on standard plastic culture, but tend to differentiate spontaneously toward all three germ layers. Mouse, rat, and human ES cells require distinct culture conditions for the maintenance of their pluripotency state. A feeder layer or different growth factors, cytokines, and small molecules are routinely used to counteract spontaneous differentiation and to promote ES cell self-renewal.

Current protocols for mouse embryonic stem (mES) cell expansion require feeder cells or matrices from animal sources which have been the major obstacle to obtain clinically significant cells due to safety issues (e.g., pathogen contamination, immunogenicity of the feeder layer), difficulty in quality control, and high expense. These protocols involve growing cells on mouse embryonic fibroblast feeder cells or on gelatin in media supplemented with fetal bovine serum (FBS) and interleukin-6 (IL-6) family member cytokines such as the leukemia inhibitory factor (LIF) [6].

A great challenge lies in identifying simple, repeatable, cost-effective, and optimum substrates capable of replacing mouse or human feeder cell layers with feeder- and serum-free culture conditions. For example, extracellular matrix (ECM) based on the conditioned medium of human fibroblasts under serum- and xeno-free culture conditions [21], or conditioned medium in a feeder-free environment [28, 30]. Other strategies have involved surface functionalization [23] or three-dimensional (3-D) matrices [1].

Although many important regulating mechanisms of ES cells have been revealed by means of two-dimensional (2-D) culture systems [11, 17], flat culture substrates are far from replicating the 3-D environment that cells experience in situ. Indeed, mES cultured in 2-D systems experience a surrounding microenvironment that is hardly comparable to the 3-D niche in vivo. Several environmental cues have been shown to regulate adhesion, migration, self-renewal, and differentiation of ES cells [1, 4, 9, 10]. For example, surface topography and 3-D geometry have been proven to maintain long-term ES cells pluripotency by inhibiting cell spreading, which makes the cells less flat thus increasing the clone integrity [2, 31].

Most of the currently available approaches for 3-D scaffold fabrication are based on self-assembly methods, and do not accurately control the geometrical structure of the substrate, which might play a crucial role in the determination of the stem cell fate [14].

A novel technology which overcomes these limitations is laser two-photon polymerization (2PP) [13]. This is a maskless direct laser writing technique that enables arbitrary microarchitectures to be manufactured with a spatial resolution down to 100 nm, thus better than the light diffraction limit. In 2PP, photopolymerization occurs by nonlinear two-photon absorption induced by femtosecond laser pulses in transparent materials. Besides active research into the synthesis of new, biocompatible and biodegradable materials [19, 29], most research groups have used hybrid inorganic-organic resins because they provide an excellent compromise between ease of use and mechanical robustness of the fabricated structures [20]. The biocompatibility of these materials has also been extensively demonstrated [5, 12, 24]. In our previous works, we developed structurally biomimetic 3-D synthetic niches, or “nichoids”, for mesenchymal stem cell (MSC) culture [15, 16, 25, 26]. These nichoids are able to counteract cell migration between adjacent areas of the substrate, thus exerting a physical containment on cells by their microarchitecture. We observed evidence of spontaneous lineage commitment in monolayer culture surrounding the nichoids, but not inside them, suggesting that the nichoids were able to direct stem cell homing, proliferation, and multipotency maintenance.

In this study we investigate whether these 3-D nichoid substrates are also able to preserve cell pluripotency in ES cells. We thus designed and fabricated a scaled-up version of the nichoid substrate with approximately 168 blocks of niches per sample, covering most of the available culture surface. This greatly improved layout enabled us to obtain a higher number of nichoid-cultured cells compared to our previous studies [15, 16, 25, 26] and to minimize the interference effects from cells cultured on the flat substrate surrounding the niches. The effect of the nichoid substrate on mES pluripotency maintenance and differentiation, in the absence of a feeder layer and LIF, was studied via immunofluorescence image colocalization and quantification of specific pluripotency and differentiation markers.

## Methods

### Preparation of the nichoid culture substrate by two-photon laser polymerization

**Fig. 1:**
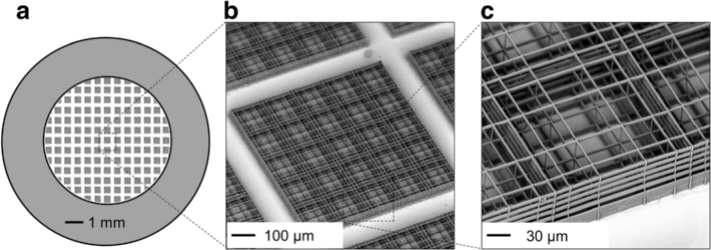
The nichoid culture substrate fabricated by two-photon laser polymerization (2PP). **a** The nichoid substrate, consisting of 168 squared blocks made up of 5 × 5 individual nichoids, laser written on a glass coverslip. To prevent cell adhesion on the flat substrate surrounding the surface covered by the nichoid blocks, a poly-dimethylsiloxane (PDMS) ring with a 7-mm inner diameter was UV-bonded to the glass coverslips (*gray*). **b** SEM of a nichoid block. **c** Detail of a single nichoid, which shares the lateral confinement walls with the neighboring nichoids of the 5 × 5 block

The nichoids were fabricated by 2PP in the SZ2080 photoresist [18] with 1 % concentration of Irg photoinitiator (Irgacure 369, 2-Benzyl-2-dimethylamino-1-(4-morpholinophenyl)-butanone-1). The laser used for 2PP was a cavity-dumped Yb:KYW system [8] producing pulses with 300-fs duration and 1-MHz repetition rate at 1030 nm wavelength, focused with a 1.4 numerical aperture (NA) oil immersion objective (Plan-APOCHROMAT, 100× oil immersion, Carl Zeiss, Oberkochen, Germany). Optimum fabrication conditions were 1.5 mm/s writing speed, 12 mW average power (before the objective). Computer-controlled, three-axis motion stages (ANT130, Aerotech, Pittsburgh, PA, USA) were used to translate the sample relative to the laser focal spot to form the desired microarchitectures. A total of 168 blocks of niches (or "nichoids") were laser written directly onto circular glass coverslips of 150 μm thickness and 12 mm diameter (BioOptika, Milan, Italy). To avoid cell adhesion on the flat substrate surrounding the surface covered by the nichoid blocks, a poly-dimethylsiloxane (PDMS, Sylgard, Dow Corning, Midland, MI, USA) ring with a 7-mm inner diameter was UV-bonded to the glass coverslips. Since PDMS prevented cell proliferation, the resulting surface available for cell culture consisted in a circular area of 7 mm in diameter (Fig. 1a).

Each nichoid block was made up of 25 repetitive niche units, 30 μm high and 90 μm × 90 μm in transverse dimensions, consisting of a lattice of interconnected lines, with a graded spacing between 10 and 30 μm transversely, and a uniform spacing of 15 μm vertically (Fig. 1b). In a previous study, this geometry was found to be able to maximize mesenchymal stem cell (MSC) homing and proliferation [25].

The overall size of each nichoid block was 30 μm high and 450 μm × 450 μm in transverse dimensions. The spacing between nichoid blocks was set to 80 μm (Fig. 1b, c). Each niche, as well as each 5 × 5 nichoid block, was surrounded by four outer confinement walls formed by horizontal lines spaced by 5 μm, resulting in gaps of 1 μm (Fig. 1c).

The fabricated nichoid substrates were characterized by scanning electron microscopy (SEM) (Phenom Pro, Phenom-World, Eindhoven, Netherlands). All observations were carried out at 5 kV. The cell-populated nichoid substrates were characterized by scanning electron microscopy (SEM) (EVO 50 EP, Carl Zeiss, Oberkochen, Germany). The thickness of the gold coating layer prior to SEM investigation was in the range of 15–20 nm. All observations were carried out at 2 kV.

### Preparation of kidney substrate by decellularization

Healthy male Sprague-Dawley rats (Charles River S.p.A., Calco, Italy) weighing 250–400 g were used. After anesthesia with isoflurane, the left kidney was retrieved by a well-established surgical procedure [3]. The kidney was initially perfused with a solution of 1 % sodium dodecyl sulfate (SDS, Sigma-Aldrich Co. LLC, St Louis, MO, USA) in distilled water through the arterial cannula for 6 hours at a flow rate of 0.4 ml/minute. Decellularized scaffolds were then gently washed with distilled water and perfused by phosphate-buffered saline (PBS, Life Technologies Italia, Monza, Italy) containing 1 % penicillin/streptomycin (Life Technologies Italia) to remove all the cellular debris and chemical residues.

All animal studies were approved by the Institutional Animal Care and Use Committees of the Mario Negri Institute, Milan, Italy. Animal care and treatment were conducted in accordance with the institutional guidelines, in compliance with national (DL no. 26, G,U, no. 61, 2014) and international laws and policies (Dir. 2010/63/EU, 9/22/2010).

### Murine ES cell culture

Due to limitations of the national law on the use of human ES cells, we used murine ES (mES) cells. The R1 mES cells were obtained through a material transfer agreement (MTA) from A. Nagy laboratory (Mount Sinai Institute, Toronto, ON, Canada). R1 mES cells were cultured and passaged every 2 days on mitotically inactivated mouse embryonic fibroblasts (MEFs; E13.5; strain 129) in proliferation medium DMEM supplemented with 1 mM sodium pyruvate, 100 μM nonessential amino acids, 2 mM L-glutamine, 100 U/ml penicillin–100 μg/ml streptomycin, 0.1 mM β-mercaptoethanol, 10 % ES cell-qualified FBS (all products were obtained from Life Technologies Italy), and 1000 U/ml LIF (Merck Millipore, Billerica, MA, USA).

### Substrate preparation and cell culture

The 2PP-patterned substrates and glass controls were washed thoroughly, kept for 20 minutes in deionized water, disinfected for 1.5 hours in 70 % ethanol, washed repeatedly in sterile deionized water, dried and UV-sterilized. Kidney substrate was frozen in liquid nitrogen, cut into 30-μm sections and sterilized as previously described [3]. Each sample was positioned inside a well of an Ultra-Low Attachment 24 multi-well plate (Costar 3473, Corning, Corning, NY, USA). After detachment, mES cells were suspended in complete medium and seeded in the wells, at a density of 10,000 cells/cm^2^ in the absence of a feeder layer and with LIF conditioning until day 3. The medium was then replaced with culture medium devoid of LIF, and freshly replaced every 2 days for 14 days. Cell morphology and embryoid body (EB) size was monitored and assessed during the culture using a standard inverted microscope (Axiovert 40 C, Carl Zeiss Inc., Göttingen, Germany) equipped with a digital camera (PowerShot G5, Canon Inc., Tokyo, Japan).

After each independent experiment, mES cells were detached by trypsin-EDTA (0.5–0.2 g/L; Invitrogen, Carlsbad, CA, USA) and the nichoids were then washed thoroughly, kept in deionized water, disinfected for 1.5 hours in 70 % ethanol, washed repeatedly in sterile deionized water, dried, UV-sterilized, and re-seeded following the protocol described above.

### Immunofluorescence staining

After 3, 7, and 14 days in culture, cells on different substrates were fixed in a 2 % paraformaldehyde (Società Italiana Chimici, Rome, Italy) and 4 % sucrose (Sigma-Aldrich) solution for 10 minutes at room temperature. Cells were then permeabilized in 0.1 % triton X-100 (Sigma-Aldrich) and treated with 3 % bovine serum albumin (BSA) (Sigma-Aldrich) for 1 hour at room temperature. Immunofluorescence staining for octamer-binding transcription factor 4 (OCT4) was performed to evaluate the stemness preservation of the cells cultured on the nichoid, the flat glass, and the kidney substrate. In addition, mES cells were stained for GATA-binding protein 4 (GATA4) and SOX-17 to assess the differentiation toward the endoderm germ layer. NKX2-5, α-smooth muscle actinin (α-SMA) and the matrix proteins collagen I and osteocalcin were stained for the mesodermal differentiation. To evaluate the ectodermal differentiation, mES cells were stained by βIII-tubulin.

Cells were incubated with Cy3-labeled mouse monoclonal anti α-SMA (diluted 1:100; Sigma-Aldrich) or Alexa Fluor® 488-labeled mouse monoclonal anti βIII-tubulin (diluted 1:100; Millipore, Temecula, CA, USA). Cells were also incubated with mouse monoclonal anti-NKX2-5 (diluted 1:250; Thermo Fisher Scientific, Waltham, MA, USA), or mouse monoclonal anti-GATA4 (diluted 1:100; Santa Cruz Biotechnology, Inc., Dallas, TX, USA), or mouse monoclonal anti-SOX17 (diluted 1:100; Santa Cruz Biotechnology, Inc), or mouse monoclonal anti-osteocalcin (diluted 1:100; Jackson ImmunoResearch Europe Ltd., Newmarket, UK) or mouse monoclonal anti-collagen I (diluted 1:2000; Jackson ImmunoResearch Europe Ltd.) or mouse monoclonal anti-Oct-3/4 (diluted 1:50; Santa Cruz Biotechnology, Inc.) followed by incubation with donkey anti-mouse IgG Cy3 conjugate (diluted 1:100; Jackson ImmunoResearch Europe Ltd.) or goat anti-rabbit FITC conjugate (diluted 1:25; Jackson ImmunoResearch Europe Ltd.). Filamentous actin (F-actin) was stained by phalloidin (diluted 1:40; Invitrogen). Counterstaining with DAPI (1 μg/ml, Sigma-Aldrich) was performed for cell nuclear staining. Finally, samples were mounted with a fluorescent mounting medium (Dako Cytomation, Carpinteria, CA, USA) and immunofluorescence images were acquired by laser confocal microscopy (LSM 510 Meta, Carl Zeiss, Jena, Germany).

### Immunofluorescence image processing and quantification

To quantify the co-occurrence of the embryonic stemness or the differentiation nuclear markers and the DAPI-stained nuclei, 15 Z-stacks of fluorescent images were acquired for each channel and for each experimental group at days 3, 7, and 14. The parameters were kept constant in each acquisition. The Z-stack channels were split and processed as follows: (i) a denoise step consisting of the subtraction of the background; specifically, for the nichoid Z-stacks, a further step at this stage was needed to remove the autofluorescent structure by mask subtraction. Then, (ii) a segmentation step, which identified the regions of interest (ROIs) (i.e., the DAPI-stained nuclei and the nuclear marker), by setting a manual threshold (Additional file 1: Figure S1a). Finally, (iii) a measurement step on the resulting 8-bit Z-stacks was performed by a custom-made script (ImageJ 1.43, National Institute of Mental Health, Bethesda, MD, USA). For both the DAPI and the nuclear marker channels, we measured the nuclear area fraction given as the percentage ratio between the average surface occupied by the nuclear marker and the total surface of the DAPI-stained nuclei. We also measured the maximum and minimum intensities to prevent saturated images.

Concerning the quantification of cytoplasmic markers (i.e., α-smooth muscle actinin), we adopted the above-mentioned procedure (Additional file 1: Figure S1b) and measured the total surface area in square pixel.

To assess the EB size, the Feret diameter was then measured directly onto the acquired phase contrast images. Finally, cell count per EB was performed on images acquired in fluorescence on the DAPI-labeled samples.

### Embryoid body fire rate analysis

Beating EB time-lapse videos were recorded using a standard inverted microscope (Axiovert 40 C, Carl Zeiss Inc., Göttingen, Germany) equipped with a digital camera (PowerShot G5, Canon Inc.), imported in ImageJ (ImageJ 1.43, National Institute of Mental Health) and converted into 8-bit grayscale. For each set of time-lapse images, ROIs including the beating colonies and the surrounding cells were manually drawn and kept fixed in position and dimensions throughout the time analysis. By drawing a line crossing the ROIs (i.e., crossing both the beating and the neighboring non-beating cells), we then monitored changes in the gray intensity profile along the Z-axis. This resulted in an estimation of the contractility frequency of the EBs.

### Statistical analysis

Three independent experiments (*N* = 3) on nichoid samples, flat glass substrates and kidney matrices were performed. For each group, n = 15 fields were acquired at days 3, 7, and 14. Mean values and standard deviations were computed, and the groups were compared using one-way analysis of variance (ANOVA) for independent samples with OriginPro software (OriginLab Corporation, Northampton, MA, USA). Pairwise comparisons among the individual samples were developed through Tukey’s HSD test on the ANOVA groups. Discrepancies among groups were considered significant if the *p* value was < 0.05.

## Results and discussion

We successfully fabricated 168 nichoids directly onto 12-mm diameter standard microscope glass slides, covering 70 % of the available culture surface (Fig. 1a). The scan speed and the laser power (1.5 mm/s and 12 mW, respectively) were optimized for the best mechanical integrity of the nichoids and to reduce the amount of microfabrication time (i.e., 11–12 hours to pattern 70 % of the available culture surface) (Fig. 1b, c).

**Fig. 2:**
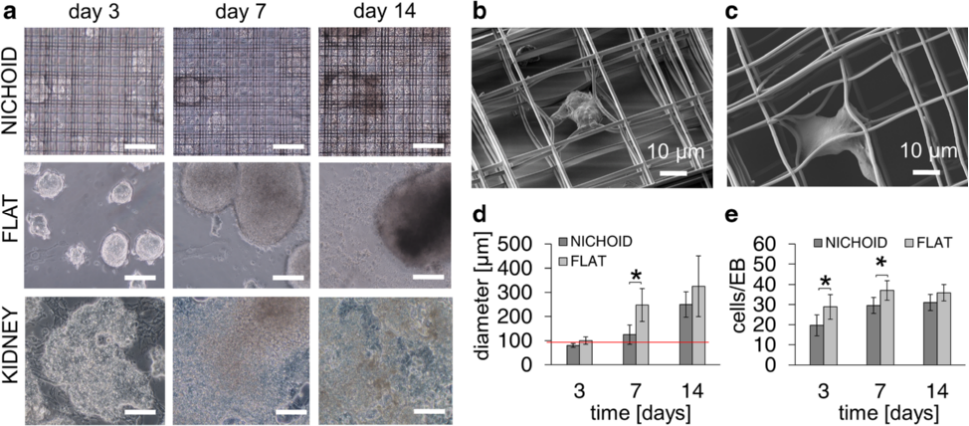
Morphology of the embryoid bodies formed by mES cells cultured in the nichoid substrates, compared to flat glass and to kidney ECM. Cells were cultured in the absence of a feeder layer and with LIF up to day 3, then without either a feeder layer or LIF from days 4 to 14. **a** Phase contrast images at days 3, 7, and 14. The scale bar is 100 μm. **b**, **c** SEM images of an embryoid body adhering to the nichoid. **d** Embryoid body diameter; *n* = 15, ^*^ *p* < 0.01. The *red line* shows the dimension of an individual niche. **e** Number of cells per embryoid body; *n* = 15, ^*^ *p* < 0.01

Our first aim was to confine the mES cells within the nichoids. We expected that, upon cell seeding, a major fraction of the cells would fall by sedimentation driven by gravity inside the nichoids and that the confinement walls would prevent these cells from leaving the nichoids during culture. However, a small fraction of cells anchored themselves onto the 80-μm gap flat glass surface in between the nichoids. Interestingly, mES cells adhered to the nichoid substrates in the absence of a feeder layer, thus demonstrating that these nichoids provide favorable conditions for cell adhesion (Fig. 2b). mES cells, maintained in culture with LIF conditioning up to day 3, and with neither a feeder layer nor LIF conditioning from days 4 to 14, formed EBs. While EB configuration was immediately lost for those cells cultured on kidney ECM substrates, EBs cultured on both the nichoid and the 2-D glass substrates were preserved up to day 3 (Fig. 2a). While EBs on 2-D glass substrates greatly increased in size and spread during the culture, EBs in the nichoids maintained their spherical morphology and dimension (Fig. 2a). This feature was also confirmed by SEM analysis (Fig. 2b, c) which showed EBs adhered to the mid-plane of the nichoid preserving their round configuration. We attribute such behavior to the physical and geometrical constraints provided by the nichoid architecture.

To quantify the containment effect, we measured the EB Feret diameter (Fig. 2d). While the sizes of both EBs in the nichoids and 2-D glass at day 3 were comparable (82.50 ± 7.8 μm and 100 ± 15.45 μm, respectively), the EB diameter in the nichoids was systematically lower at day 7 (120.50 ± 40.12 μm, in nichoids, 248.40 ± 68.45 μm on 2-D glass, n = 15, *p* value = 0.01) and day 14 (250.01 ± 52.35 μm, in nichoids, 325.40 ± 125.30 μm on 2-D glass). The average EB diameter in nichoids at days 3 and 7 was comparable to the characteristic length (i.e., 90 μm) of the repetitive niche units composing the nichoid substrate (Fig. 1c, Fig. 2d).

These measurements prove that there is a containment effect due to the 3-D nichoid architecture (Fig. 2d). In addition, the average cell number per EB in nichoids was significantly lower than that calculated on 2-D glass substrates at day 3 (19.70 ± 5.20 cells/EB and 28.88 ± 6.08 cells/EB, respectively, n = 15, *p* value = 0.01) and day 7 (29.55 ± 3.96 cells/EB and 37.04 ± 4.83, respectively, *n* = 15, *p* value = 0.01). Conversely, no statistical differences were found for the number of cells normalized by EB at day 14 (31.08 ± 4.05 cells/EB in the nichoids and 35.88 ± 4.08, n = 15, *p* value = 0.01) (Fig. 2e).

The number of cells per EB in the nichoids demonstrated, not only the confinement effect due to the nichoid architecture (Fig. 2d), but also a higher proliferation rate compared to the 2-D glass culture substrates (Fig. 2e). At days 7 and 14 compared to day 3, the proliferation rate measured was 50 % greater in average in nichoids, compared to 2-D flat glass substrates. However, this confinement effect diminished slightly with culture time. In fact, as the cell number increased, together with the size of the EBs over time, the available nichoid internal volume decreased. In addition, since there was no available space where they could grow, the EBs spread out from the 3-D architecture and no longer experienced the physical containment provided by the nichoid.

**Fig. 3:**
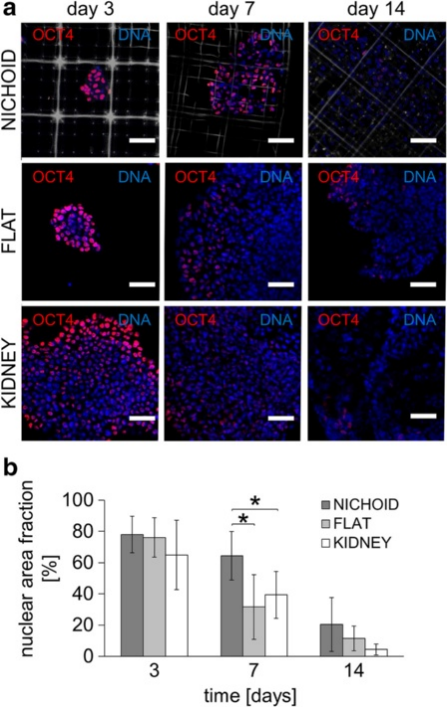
Maintenance of pluripotency of mES cells cultured in the nichoid substrates, compared to flat glass and to kidney ECM. Cells were cultured in the absence of a feeder layer and with LIF up to day 3, then without either a feeder layer or LIF from days 4 to 14. **a** Immunofluorescence for OCT4 (*red*) and DAPI (*blue*) on the nichoid (*gray*), the flat glass and the kidney ECM at days 3, 7, and 14. The scale bar is 50 μm. **b** Quantification of OCT4 expression by image processing; *n* = 15, ^*^ *p* < 0.01

Our second aim was to assess the nichoid confinement effect on EBs on pluripotency maintenance and differentiation toward the three germ layers in feeder-free and LIF-free culture conditions. To evaluate stemness promotion and inhibition to differentiation, we stained and evaluated the co-occurrence of OCT4 and DAPI. The OCT4 pluripotent marker was highly expressed in cells cultured on both EBs in the nichoids, 2-D glass and kidney substrates in the presence of LIF conditioning at day 3 (78.80 ± 11.65 %, 76.16 ± 12.52 % and 64.94 ± 22.24 %, respectively). Once the LIF was devoid, OCT4 expression turned out to be significantly greater in nichoids compared to 2-D glass and kidney ECM substrate at day 7 (64.41 ± 15.51 %, 31.65 ± 20.75 % and 39.39 ± 15.00 %, respectively) (Fig. 3a, b, *n* = 15, *p* value = 0.01). However, OCT4 was greatly diminished in all of the substrates tested at day 14 (20.42 ± 17.30 % in nichoids, 11.50 ± 7.86 % on glass and 4.34 ± 3.46 % on kidney ECM).

While such observations were to be expected for cells on kidney ECM, where EBs were rapidly lost (Fig. 2a), the pluripotency maintenance on EBs in the nichoids may have arisen from the physical constraints provided by their 3-D microarchitecture. In fact, OCT4 expression on 2-D glass substrates, in which EBs spread considerably and increased in size (Fig. 2c), greatly decreased at day 7 (Fig. 3b).

Note that at day 14, OCT4+ EBs were still greater than the EBs on 2-D glass substrates, but not significantly because EBs managed to escape from the nichoids, as can be observed from the measurement of their average diameter (Fig. 2d) and the number of cells per EB (Fig. 2e). In fact, at day 14 the EB size exceeded the repetitive niche unit characteristic length (i.e., 90 μm, red line in Fig. 2d), thus losing the physical constraint provided by the 3-D architecture. This means that EBs were not completely confined in the nichoids, at least not in the uppermost part. Thus, day 7 was the most representative time point to evaluate the effect of the nichoids on mES cell pluripotency because of the absence of LIF medium conditioning and the presence of physical containment due to the nichoids.

To further examine this effect, we plan to increase the nichoid height to prolong the time in which the EBs are constrained. However, a good compromise will be needed between the manufacturing times, the optical accessibility of the system (i.e., the focal depth of confocal or, if necessary, a two-photon microscope) and the necessity to easily detach and collect cells by trypsin at the end of the culture.

**Fig. 4:**
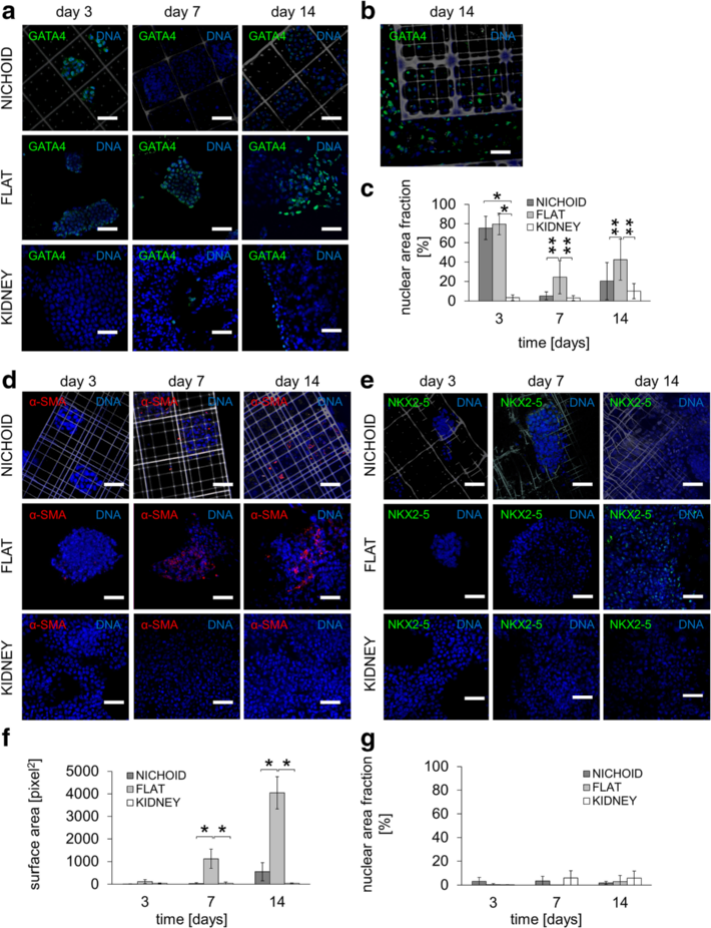
Spontaneous endodermal and mesodermal differentiation of mES cells cultured in the nichoid substrates, compared to flat glass and to kidney ECM. Cells were cultured in the absence of a feeder layer and with LIF up to day 3, then without neither a feeder layer nor LIF from day 4 to day 14. **a** Immunofluorescence for GATA4 (*green*) and DAPI (*blue*) in the nichoid (*gray*), the flat glass and the kidney ECM at day 3, 7, and 14. The scale bar is 50 μm. **b** Detail of a flat region surrounding a nichoid block, showing a possible paracrine effect generated by GATA4+ cells on the expression of the GATA4 marker by cells of peripheral nichoids. The scale bar is 20 μm. **c** Quantification of GATA4 expression by image processing; n = 15, ^*^ *p* < 0.01 ^**^ *p* < 0.05. **d** Immunofluorescence for α-SMA (*red*) and DAPI (*blue*) in the nichoid (*gray*), the flat glass and the kidney ECM at day 3, 7, and 14. The scale bar is 50 μm. **e** Immunofluorescence for NKX2.5 (*green*) and DAPI (*blue*) in the nichoid (*gray*), the flat glass and the kidney matrix substrate at day 3, 7, and 14. The scale bar is 50 μm. **f** Quantification of α-SMA expression by image processing; n = 15, ^*^ *p* < 0.01 ^**^ *p* < 0.05. **g** Quantification of NKX2.5 expression by image processing; n = 15, ^*^ *p* < .0.01 ^**^ *p* < 0.05

We thus assessed the differentiation potential toward the endoderm germ layer by staining and quantifying the co-occurrence of GATA-4 and DAPI in feeder-free layer culture conditions (Fig. 4a, c). This endoderm marker was highly expressed in cells cultured on both EBs in the nichoids and 2-D glass substrates (75.12 ± 12.33 %, 79.57 ± 11.31 %, respectively). On the other hand, it was negligible on kidney ECM (3.42 ± 2.86 %) (Fig. 4c, *n* = 15, *p* value = 0.01) in the presence of LIF conditioning at day 3. This could be explained by the dimethyl sulfoxide (DMSO) used in cell freezing. DMSO has been reported as an induction factor for endodermal differentiation [22]. In the absence of LIF, GATA4 expression in nichoids thus slowed down significantly compared to the 2-D glass (5.24 ± 3.97 %, 24.42 ± 17.07 %, respectively. *n* = 15, *p* value = 0.05) at day 7, while it was negligible on kidney ECM up to day 14. GATA4 in the nichoids and in the 2-D glass increased at day 14 (20.39 ± 19.06 %, 42.21 ± 22.15 %, respectively. *n* = 15, *p* value = 0.05), resulting in an up-down-up expression that is well documented in the literature [7] (Fig. 4a, c). However, this behavior could also be due to a possible paracrine signaling effect (Fig. 4b). In fact, GATA4^+^-EB were mostly localized at the outer boundaries of the nichoids, in particular close to the GATA4^+^ cells grown on the 80-μm gap flat glass surface in between the nichoids. Therefore, such cells experiencing the 2-D environment could have affected the mES cell differentiation in the peripheral nichoids.

We also evaluated the EB differentiation potential toward the mesoderm germ layer by staining and quantifying α-SMA, as well as the co-occurrence of NKX2-5 and DAPI in feeder-free layer culture conditions (Fig. 4d-g). The expression of α-SMA was negligible at all time points for EBs cultured in the nichoids as well as in the kidney ECM. Conversely, EBs cultured on the 2-D glass substrate were significantly upregulated with respect to the nichoids both at day 7 (1117.15 ± 425.06 pixel^2^ on 2-D glass, 36.73 ± 35.63 pixel^2^ in nichoids, *n* = 15, *p* value = 0.01) and day 14 (4045.80 ± 716.15 pixel^2^ on 2-D glass, 550.42 ± 398.02 pixel^2^ in nichoids, *n* = 15, *p* value = 0.01) (Fig. 4d, f). NKX2-5 was weakly expressed in all the culture substrates tested (Fig. 4e, g). However, NKX2-5 expression slightly increased on 2-D glass substrates at day 14. Both cardiac mesodermal differentiation markers were localized on glass substrates and therefore, this effect might be related to the stiffness of the glass substrate (of the order of GPa).

**Fig. 5:**
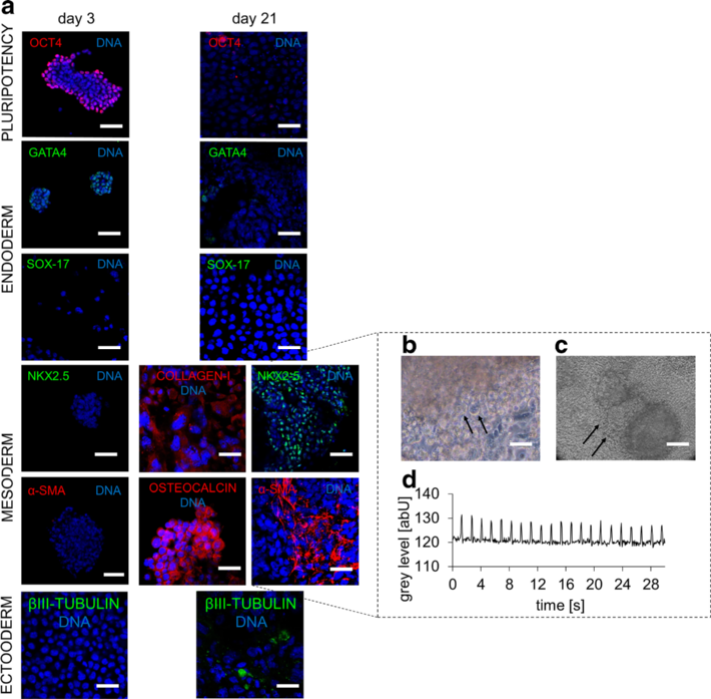
Spontaneous differentiation of mES cells cultured on flat glass substrates. Cells were cultured in the absence of a feeder layer and with LIF up to day 3, then without either a feeder layer or LIF from days 4 to 21. **a** Immunofluorescence for OCT4 (*red*), GATA4 (*green*), SOX-17 (*green*), NKX2.5 (*green*), α-SMA (*red*), collagen type I (*red*), osteocalcin (*red*), βIII-tubulin (*green*) and DAPI (*blue*) at days 3 and 21. The scale bar is 50 μm. **b** Phase contrast image of differentiated cells at day 19, showing a morphology resembling pacemaker-like cells (*arrows*). The scale bar is 100 μm. **c** Phase contrast image of beating cells (*black arrows*) surrounding two embryoid bodies at day 19 (cell beating is shown in the Additional files 2: Video 1 and Additional file 3: Video 2). The scale bar is 100 μm. **d** Quantification of the beating frequency by image processing

To assess the pluripotent nature of the cells used in this study and to adequately characterize their response in our control conditions, we investigated the spontaneous differentiation toward the three germ layers in long-term culture on 2-D glass substrates, without nichoid microstructures. This experiment was conducted in the absence of a feeder layer and with LIF conditioning up to day 3, while without feeder layer and LIF conditioning from day 4 to day 21 (Fig. 5a). Several markers were tested. As mentioned, we observed OCT4^+^ cells at day 3 (i.e., LIF conditioning and no feeder layer) and OCT^-^ EBs at day 21 (i.e., no LIF conditioning and no feeder layer). Concerning the endodermal differentiation, GATA4 showed an up-down behavior at day 3 and day 21, respectively, which is also reported in [7]. As previously mentioned, a possible explanation for this outcome may be due to DMSO reported as an induction factor for endodermal differentiation [22].

We also assessed the SOX17 expression, which turned out to be negative for the whole culture, including in the nichoids (data not shown). Interestingly, α-SMA and NKX2-5 in EBs cultured on flat substrates were negligible up to day 3, and greatly expressed at day 21. Such markers are involved in mesodermal cardiac differentiation and were consistent with the observation of beating cells surrounding the EBs at day 19. This highlighted the particular spherical morphology typical of pacemaker-like cells (Fig. 5b, arrows), together with spontaneous beating (Fig. 5c, arrows, Additional files 2: Video 1, Additional file 3: Video 2, Additional file 4: Video 3, Additional file 5: Video 4 and Additional file 6: Video 6). The measured beating frequency was 42 beats/minute (Fig. 5d). Collagen I, a nonspecific matrix differentiation marker and osteocalcin, a tardive osteogenic differentiation marker, were also expressed in EBs in long-term culture on 2-D glass. Finally, we evaluated βIII-tubulin expression to assess the ectodermal differentiation on 2-D glass substrates. As expected, the differentiation was negative at day 3 (i.e., with LIF conditioning), but surprisingly it was upregulated at day 21 (i.e., without LIF conditioning). This could be due to the FBS in culture that might contain inductive factors [27].

**Fig. 6:**
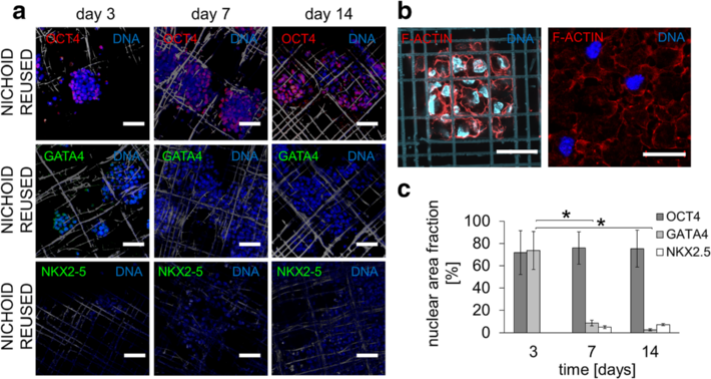
Spontaneous differentiation of mES cells cultured on reused nichoid substrates. Cells were cultured in the absence of a feeder layer and with LIF up to day 3, then without either a feeder layer or LIF from days 4 to 14. **a** Immunofluorescence for OCT4 (*red*), GATA4 (*green*), NKX2.5 (*green*) and DAPI (*blue*) at days 3, 7, and 14. The scale bar is 50 μm. **b** Immunofluorescence for F-actin (*red*) and DAPI (*blue*) showing a few residual embryoid bodies anchored both to the nichoid (*left*) and the flat glass (*right*) after trypsinization. The scale bar is 30 μm. **c** Quantification of OCT4, GATA4 and NKX2.5 expression by image processing; n = 15, ^*^ *p* < 0.01

Finally, our third aim was to assess the pluripotency maintenance and differentiation toward the three germ layers in feeder-free layer culture conditions in reused nichoids. We cultured mES cells in the absence of a feeder layer and with LIF conditioning up to day 3, then without either a feeder layer or LIF conditioning from day 4 to day 14. To evaluate stemness promotion and inhibition to differentiation, we stained and evaluated the co-occurrence of OCT4 and DAPI, while for the differentiation potential toward the endoderm and mesoderm germ layer, we stained and quantified the co-occurrence of GATA-4 and NKX2-5 on DAPI (Fig. 6a, c). Surprisingly, EBs were OCT4^+^ throughout the culture period (74.35 ± 2.70 on average), whereas both GATA4 and NKX2-5 expression were negligible. EB-GATA4^+^ was observed at day 3. As previously mentioned, a possible explanation could be the DMSO which was reported as an induction factor for endodermal differentiation [22]. The high OCT4 expression in the reused nichoids could depend on the protocol for cell detachment by trypsin. As shown in Fig. 6b (right), F-actin (red), and therefore fragments of plasma membranes were observed with no nuclei. Despite trypsin, cell residues (and/or other secreted proteins) may have remained anchored to the substrate, favoring cell adhesion, EB growth in nichoids (Fig. 6b, left), and pluripotency maintenance.

Our study revealed that the nichoid allowed expansion and pluripotency maintenance without feeder cells in the absence of exogenous soluble factors (i.e., LIF) by providing biophysical signals for pluripotency in prolonged culture of mES cells. The observation that mES cells lost EB configuration on 2-D flat glass substrates suggests that this 3-D expansion behavior was mediated by the physical microenvironment provided by the nichoid alone. Compared to 2-D glass substrates, the nichoid substrate provides cells with an increased surface-to-volume ratio and space to adhere and proliferate. In addition, the nichoid 3-D architecture, as well as its physical/geometrical constraint, may be the primary feature controlling the ES cell fate.

Our results are in agreement with the most recent literature on the effect of the biophysical environment on ES long-term pluripotency maintenance [1, 21, 23]. For example, various functionalized polymer substrates have been demonstrated to facilitate the long-term maintenance of human pluripotent stem cells (hPSCs) in a xeno-free culture and feeder-free culture system [23]. In addition, in [1] human induced pluripotent stem cells (hIPSCs) and human embryonic stem cells (hESCs) grown in a 3-D nanofiber environment maintained their pluripotency as long as they were kept on nanofibers. In contrast to these studies, we did not use a soluble conditioning medium for mES pluripotency [1, 21, 23, 28, 30].

## Conclusions

Our results show that the nichoid was the only substrate among those tested that maintained a pluripotency gene switched on and, simultaneously, three differentiation genes switched off in the prolonged culture of mES cells. These results were achieved in the absence of exogenous soluble factors, such as LIF, and without a feeder layer. The confinement effect due to the 3-D architecture of the nichoid substrate could explain this behavior, and thus could be a potent enough cue to cause a master switch in cells. We hypothesize that the nichoid microstructures induce a genetic reprogramming of cells, primarily by controlling their cytoskeletal tension.

Further studies are necessary to understand the exact mechanism by which the physical constraint provided by the nichoid architecture is responsible for this reprogramming effect. The nichoid substrate may help in understanding the mechanisms of pluripotency maintenance in vitro, while potentially cutting the costs and risks of both feed conditioning and exogenous conditioning for the industrial-scale expansion of stem cells.

## Additional files

Additional file 1:
**Figure S1.** Methods for the quantification of gene expression by processing of images acquired in immunofluorescence. (a) Quantification method of the co-occurrence of the nuclear marker (*green*) in the cell nucleus counterstained with DAPI (*blue*). (b) Quantification method of the cytoplasmic marker expressed in square pixel. (TIF 3135 kb) Video S2. mES surrounding EBs showed a spontaneous beating at day 15 when cultured on 2-D glass substrates. (WMV 12.5 mb) Video S3. mES surrounding EBs showed a spontaneous beating at day 19 when cultured on 2-D glass substrates. (WMV 7.71 mb) Video S4. mES surrounding EBs showed a spontaneous beating at day 19 when cultured on 2-D glass substrates. (WMV 7.23 mb) Video S5. mES surrounding EBs showed a spontaneous beating at day 20 when cultured on 2-D glass substrates. (WMV 7.54 mb) Video S6. mES surrounding EBs showed a spontaneous beating at day 20 when cultured on 2-D glass substrates. (WMV 6.33 mb)

## Abbreviations

2-D: Two-dimensional
2PP: Two-photon polymerization
3-D: Three-dimensional
ANOVA: Analysis of variance
BSA: Bovine serum albumin
EB: Embryoid body
ECM: Extracellular matrix
ES: Embryonic stem
GATA4: GATA-binding protein 4
hESC: Human embryonic stem cell
hiPSC: Human induced pluripotent stem cell
IL-6: Interleukin-6
LIF: Leukemia inhibitory factor
mES: Mouse embryonic stem
MSC: Mesenchymal stem cell
NA: Numerical aperture
OCT-4: Octamer-binding transcription factor 4
PDMS: Polydimethylsiloxane
ROI: Region of interest
SEM: Scanning electron microscopy
α-SMA: α-smooth muscle actinin
