# Supplementary figures and images for: Two-photon polymerized “nichoid” substrates maintain function of pluripotent stem cells when expanded under feeder-free conditions
Source: Stem Cell Res Ther. 2016 Sep 9;7(1):132. doi: 10.1186/s13287-016-0387-z (PMC5016857; doi:10.1186/s13287-016-0387-z)

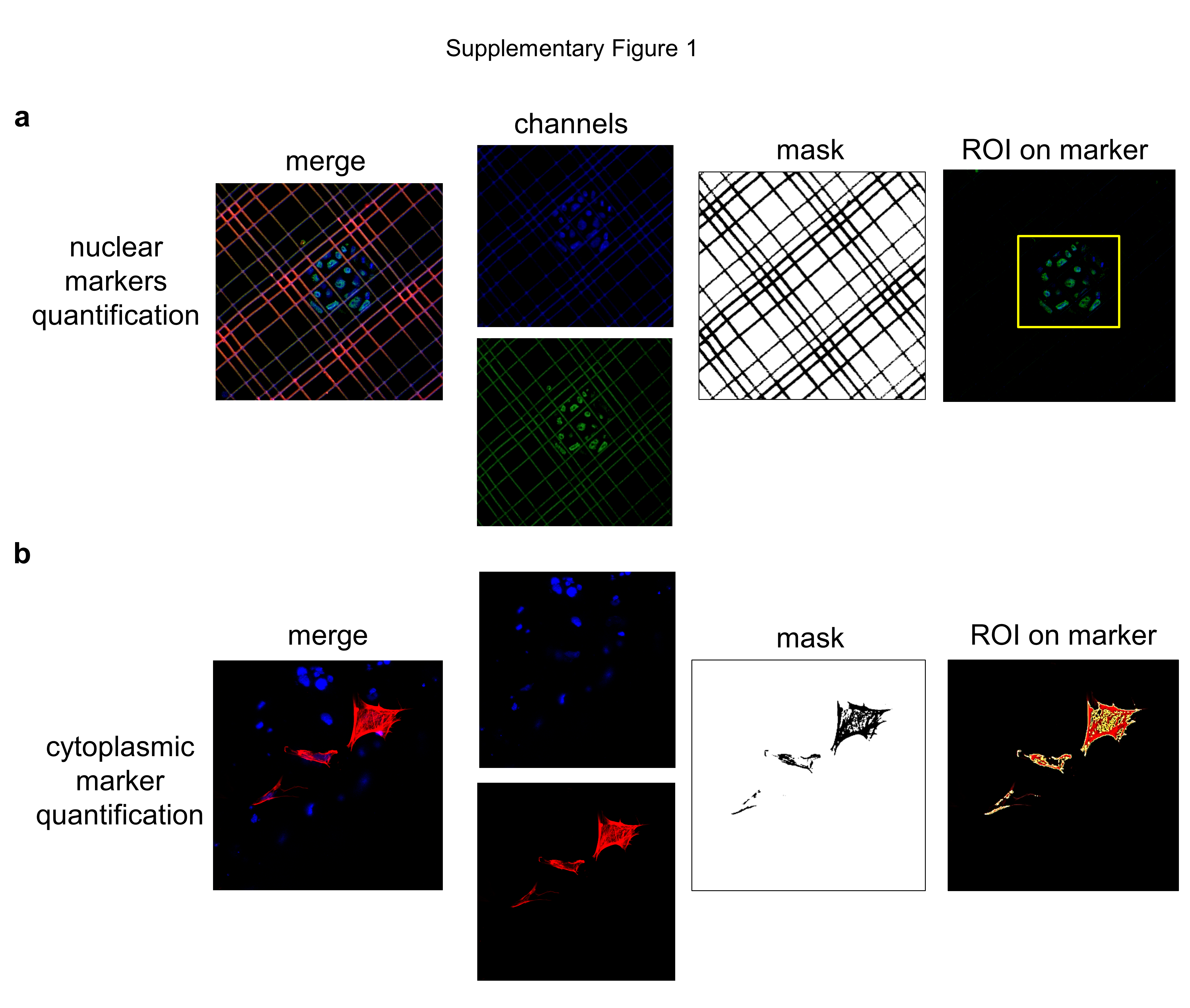

Supplement: Additional file 1: — Figure S1. Methods for the quantification of gene expression by processing of images acquired in immunofluorescence. (a) Quantification method of the co-occurrence of the nuclear marker (green) in the cell nucleus counterstained with DAPI (blue). (b) Quantification method of the cytoplasmic marker expressed in square pixel. (TIF 3135 kb) [file 13287_2016_387_MOESM1_ESM.tif]
